# Biological traits of *Quadrastichus mendeli* (Hymenoptera, Eulophidae), parasitoid of the eucalyptus gall wasp *Leptocybe invasa* (Hymenoptera, Eulophidae) in Thailand

**DOI:** 10.1051/parasite/2019008

**Published:** 2019-02-22

**Authors:** Benjakhun Sangtongpraow, Kosol Charernsom

**Affiliations:** 1 Department of Entomology, Faculty of Agriculture, Kasetsart University Bangkok 10900 Thailand

**Keywords:** Biological control, Parasitoid, *Quadrastichus mendeli*, Eucalyptus gall wasp, *Leptocybe invasa*, Hymenoptera

## Abstract

*Quadrastichus mendeli* Kim & La Salle, a parasitoid of *Leptocybe invasa* Fisher & La Salle, is a uniparental species. This study assessed the biological traits of *Q. mendeli* in the laboratory at a temperature of 27 ± 1 °C. Diets had a highly significant effect on the mean longevity of female *Q. mendeli*. Feeding honey solution prolonged the mean longevity of the parasitoid to 4.80 days. The estimated 50% survival period was 3 days. The mean potential fecundity in all ages was 8.85 eggs per female. Age had a highly significant effect on the mean egg load. There was a positive relationship between egg load and female size. The mean of realized fecundity throughout the life span was 2.47 progenies per female. The mean developmental time of *Q. mendeli* from the egg to adult stage was 27.06 days. The shorter developmental time of *Q. mendeli* in comparison to its host can be considered a reason for the successful control of *L. invasa* in Thailand.

## Abstract (fr)

*Quadrastichus mendeli* Kim & La Salle, un parasitoïde de *Leptocybe invasa* Fisher & La Salle, est une espèce uniparentale. Cette étude a évalué les traits biologiques de *Q. mendeli* en laboratoire à une température de 27 ± 1 °C. Les régimes ont un effet très significatif sur la longévité moyenne de *Q. mendeli* femelle. L’alimentation avec une solution de miel a prolongé la longévité moyenne du parasitoïde à 4,80 jours. Le temps de survie estimé à 50 % était de 3 jours. La fécondité potentielle moyenne à tous les âges était de 8,85 œufs par femelle. L’âge avait un effet très significatif sur la charge moyenne en œufs. Il y avait une relation positive entre la charge en œufs et la taille de la femelle. La fécondité moyenne réalisée au cours de la vie était de 2,47 descendants par femelle. Le temps de développement moyen de *Q. mendeli* du stade œuf au stade adulte était de 27,06 jours. Le temps de développement de *Q. mendeli,* plus court que celui de son hôte, peut être considéré comme une raison du succès du contrôle de *L. invasa* en Thaïlande.

## Introduction

*Eucalyptus camaldulensis* Dehnh. (Myrtales: Myrtaceae) is a major fast-growing hardwood tree species in Thailand, and it is used in the production of timber, pulp, bioenergy and other minor products. The cultivated areas under the cover of eucalyptus in Thailand are mainly in the east, north-east and west of the country, and their increase has given rise to eucalyptus insect pests [37]. The eucalyptus gall wasp, *Leptocybe invasa* Fisher & La Salle, originates from Australia and has become a global pest in eucalyptus plantations [32]. *Leptocybe invasa* has expanded to more than 39 countries in Asia, Africa, Europe and the Americas [36]. In Thailand, this eucalyptus gall inducer has established itself successfully in *E. camaldulensis* plantations for more than a decade [5]. It damages newly developed leaves and young twigs of *Eucalyptus* spp., and forms typical galls in the form of distinct swellings on leaf midribs, petioles and young twigs, in both plantations and nursery stock. Heavy galling causes deformity of leaf shape, abnormality of sapling growth, reduction of wood yield [32], and a subsequent decrease in farming revenue.

*Leptocybe invasa* is a thelytokous species [32, 36, 54] in which the mothers produce only diploid daughters from unfertilized haploid eggs and offspring are genetically identical to their mothers [32, 50]. The female *L. invasa* is also a pro-ovigenic species [43, 53]. Studies on the *L. invasa* population in Italy, Tunisia and Argentina found only females, indicating that *L. invasa* reproduced by thelytokous parthenogenesis [1, 9, 32, 48]. Afterwards, males were reported in Turkey [16], India [22], China [6], Taiwan [47] and Thailand [43]. Male *L. invasa* were recorded at 0.5% of the Turkish population [16] and up to 18–48% of the Chinese one [30]. The morphology of *L. invasa* was not different in the Turkish and Chinese population [36].

*Rickettsia* infection (an endosymbiotic bacterium) in the reproductive tissue induced thelytoky in *L. invasa* [36]. However, the presence of males in a thelytokous population of *L. invasa*, where *Rickettsia* was involved in the manipulation of the reproduction, could be due to the interaction between the host genotype and its own *Rickettsia* [24, 36, 50]. On the other hand, the density of *Rickettsia* could decrease at high temperatures, which caused the presence of males in the thelytokous species [3, 17, 18, 49]. Moreover, studies based on molecular and phylogenetic analyses of *L. invasa*, genetic differences at the strain level of *Rickettsia* symbionts, and sex ratio differences suggest separation of Turkish and Chinese lineages, which can therefore be treated as putative species [36]. Besides sex ratio, other biological features of *L. invasa* have been reported [8, 43, 55]. The results varied considerably with methodologies and experimental condition settings. Outbreaks of *L. invasa* have also been associated with its reproductive biology [11, 54].

The management methods used to control *L. invasa* include chemical control [26], breeding and selection of resistant planting stock [12, 29, 40], and biological control [10, 28, 35]. Chemical control is not widely accepted due to its varying success, negative effects on biodiversity, and environmental pollution [52]. As *E. camaldulensis* has a wide range of resistance to gall inducers [29], breeding and selection of resistant planting stock are carried out against *L. invasa*. Thai researchers were able to produce various eucalyptus clones [34]. The reasons for clone production were mainly to increase wood yield and resistance to eucalyptus pests. In fact, the clones vary in degrees of damage caused by *L. invasa*, and their variations in biomass were observed in different planting sites [2]. The collenchyma cell wall thickness of abaxial and adaxial ground tissues of the midrib and petiole indicate *L. invasa* resistance [2]. It also was noted that a tolerant trait from a mother clone may not be transferred to the new generation. A group of Thai researchers were able to produce a eucalyptus hybrid (*E*. *camaldulensis* Dehnh. x *E. pellita* F. Muell.) that tolerated this pest. Further attempts are being made to solve the problem of a specific planting site for this hybrid [40].

Biological control is an attractive alternative to other control methods, due to its ecological and economic benefits [7]. It is an important tool to minimize the adverse effect of *L. invasa* and limit the populations of gall inducers [14, 28]. Some parasitoids of *L. invasa* have been reported in many countries [13–15, 25, 28, 33, 35, 38, 39, 41, 51, 52], in which the parasitism level was different. The local *Megastigmus thitipornae* Dogănlar & Hassan (Hymenoptera: Torymidae) had less parasitic capacity to control *L. invasa* in Thailand [42]. The authors of this study observed a parasitoid of *L. invasa* in eucalyptus plantations. The specimens were sent to Dr. John La Salle, CSIRO, Australia, in November 2014, and he confirmed their identity as *Quadrastichus mendeli* Kim & La Salle (Hymenoptera: Eulophidae), which is a parasitoid native of Australia. From preliminary population samplings by the authors, the spread and naturally established adult of *Q. mendeli* in eucalyptus plantations was notable in some provinces of Thailand.

*Quadrastichus mendeli* is a uniparental species in which the males are unknown [28, 52]. The reproductive mode of *Q. mendeli* is thelytoky [21, 28]. A thelytokous population can grow more and faster than a sexual one [31, 45]. The thelytoky of *Q. mendeli* is due to *Rickettsia* infection [21]. The spread and natural establishment of *Q. mendeli* [35, 52] and its biological aspects such as longevity, fecundity, and development of *Q. mendeli* have been reported [28]. In general, a shorter lifespan of the parasitoid in comparison with that of its host is a desirable characteristic in successful biological control, as it can produce its progeny at a faster rate than the host, and parasitize the host population in a shorter time [19, 20, 44].

In order to reduce the use of chemicals in the agroecosystem, the study on potentiality of *Q. mendeli* to minimize damage caused by *L. invasa* should be carried out in Thailand, particularly on a susceptible clone of *E. camaldulensis*. Furthermore, the environmental conditions in the tropical region are significantly different from other areas. This makes the study worthwhile, particularly since *Q. mendeli* may be an effective control agent for *L. invasa* in this country. This research assessed the biological traits of *Q. mendeli*. Biological features studied include longevity and survival pattern, potential and realized fecundity, and developmental time. The results obtained from this research could be used to assess the potentiality of *Q. mendeli* in order to minimize the damage caused by *L. invasa* in Thailand.

## Materials and methods

### Assessment of longevity and survival pattern of *Q. mendeli*

Adult *Q. mendeli* (<6 hour-old after emergence) were sampled from leaf galls collected from an area planted with a susceptible *E. camaldulensis* clone (CT 76 clone), put in a vial (4.5 cm in diameter, 8 cm high), and fed with three different feeding treatments: no diet, pure water, and honey solution (honey:water = 1:1). Each treatment comprised five replicates and ten parasitoids per replicate. Diets of pure water and honey solution were dropped onto small cotton balls and refreshed daily until all of the wasps had died. Each vial was covered with fine mesh to provide ventilation. The number of dead and living parasitoids in each treatment was counted daily to determine the longevity and survival pattern under each diet regime. The experiments were maintained in the laboratory at a temperature of 27 ± 1 °C and 75 ± 5% relative humidity (RH). This temperature represented the average for outdoors during the period of this study. All experiments carried out in this research were also maintained at this temperature. The results from this temperature could provide more realistic data for Thailand. The longevity of *Q. mendeli* was analyzed via an *F*-test, using a statistical software program (SPSS for Windows version 11.5). Tukey’s honestly significant difference test was used to compare the means of treatment. A difference of means was considered significant at a level of *p* < 0.05.

### Determination of *Q. mendeli* fecundity

#### Potential fecundity

Potential fecundity (egg load) is expressed as the maximum number of mature eggs in the ovary that can be laid potentially by an adult female parasitoid at the time of investigation. The eggs can be counted directly by dissecting the ovary of the adult. Newly emerged female parasitoids were separated into seven vials, each covered in fine mesh, with ten parasitoids per vial. They were fed with honey solution dropped onto small cotton balls in the vials. The females were sacrificed by freezing at ages of < 6, 6, 12, 24, 48, 72, and 96 h after emergence. Their ovaries were dissected in saline solution under a stereoscope. The number of mature eggs in each female was determined. The effect of age on the mean egg load was analyzed via the *F*-test. The length of the female hind tibia of all ages was measured and used as a substitute for female size [27]. The relationship between the number of mature eggs (egg load) and size (hind tibia length) was analyzed via simple linear regression, using a statistical software program (SPSS for Windows, version 11.5).

#### Realized fecundity

This term denotes the numbers of progeny produced by female wasps that survive to emerge as adult [4]. In general, fecundity is determined by counting the real number of eggs deposited on a host per day until the female dies, and then calculating the total number of eggs laid on the host over the female’s life-time. However, *Q. mendeli* eggs were difficult to detect and count because they were laid inside leaf-galls of their hosts. Thus, this research determined the realized fecundity of *Q. mendeli* by counting the total number of emerging progeny that survived to adulthood, and expressing in terms of progeny per female [23, 46]. This study was divided into two parts as described below.

Part 1 – determination of appropriate gall stage for parasitism: the eucalyptus saplings were planted in plastic pots and kept in fine mesh cages to prevent gall wasps from entering from outside. Twenty shoots of *E. camaldulensis* saplings were sampled. Each shoot was enveloped in fine mesh, with adult *L. invasa* females (<6 hour-old after emergence) being released inside the envelope for 24 h, using three females per shoot. The development of *L. invasa* in leaf galls was divided into four stages (egg, young larva, mature larva, and pupa) and five shoots were used in each stage. The stages of *L. invasa* development were assessed in terms of number of days after *L. invasa* oviposition; egg (11 days), young larva (18 days), mature larva (30 days), and pupa (38 days) [43].

When the development of *L. invasa* reached each stage, the galled shoots were enveloped in fine mesh again. A single *Q. mendeli* (24 hour-old) was fed with honey solution before introduction to and placement on each infested shoot for 24 h. The fine mesh was removed from the shoot after the exposure. The galls of each shoot were cut and put into a vial (4.5 cm in diameter, 8 cm high) before the emergence of *L. invasa* and *Q. mendeli*. The number of emerging wasps from the galls per shoot was determined. The appropriate gall stage for the parasitism of *Q. mendeli* was assessed. The experiments were carried out in ventilated cages (1.60 m × 1.60 m × 2.0 m) covered with fine mesh and maintained in the laboratory at a temperature of 27 ± 1 °C.

Part 2 – assessment of realized fecundity: 150 shoots were sampled from *E. camaldulensis* saplings in pots. Each shoot was wrapped with fine mesh. The adult female *L. invasa* (<6 hour-old after emergence) were released inside the envelope for 24 h, using three females per shoot. As the galls reached the appropriate stage for parasitism (mature larva; 30 days from the results of Part 1), one *Q. mendeli* (24 hour-old), which had been fed with honey solution prior to use, was introduced to each infested shoot for 24 h. The parasitoid was transferred to a newly infested shoot on the following day and left for 24 h. This procedure was conducted daily until the parasitoid had died. The parasitoid was fed during the experiments with honey solution on small cotton balls hanging on the shoots. These studies were conducted in a ventilated greenhouse covered with fine mesh. The galls of each shoot were cut and put into a vial before the emergence of *L. invasa* and *Q. mendeli*. The numbers of emerging *L. invasa* and *Q. mendeli* were counted daily until no wasps emerged, and the realized fecundity of *Q. mendeli* was then assessed.

### Study on the developmental time of *Q. mendeli*

*Eucalyptus camaldulensis* saplings were planted in plastic pots and kept in fine mesh cages to prevent gall wasps entering from outside. Twenty shoots of *E. camaldulensis* were sampled. Each shoot was enveloped with fine mesh and adult female *L. invasa* (<6 hour-old after emergence), which had been fed with honey solution prior to use, were released inside the envelope for 24 h, using five females per shoot. After reaching the appropriate gall stage for parasitism, one *Q. mendeli* (24 hour-old), which had been fed with honey solution prior to use, was introduced to each infested shoot for 24 h. This study was conducted in ventilated cages (1.60 m × 1.60 m × 2.0 m) covered with fine mesh. After exposure, the fine mesh was removed from each shoot. Five leaves were collected daily. The galls were removed from the leaves and dissected daily until the parasitoid emergence was completed. The fresh larvae and pupae of the parasitoid were studied and photographed.

## Results

### Longevity and survival pattern of *Q. mendeli*

**Figure 1.**
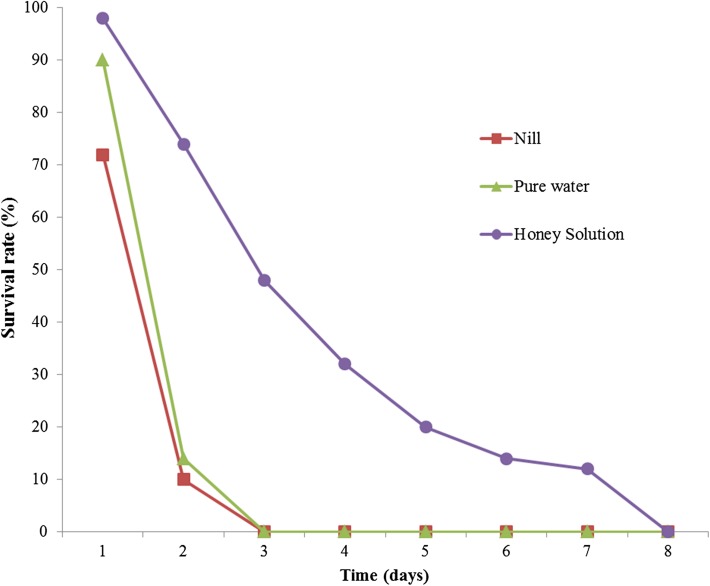
Survival patterns of female *Quadrastichus mendeli* fed on different diets.

Diets had a highly significant effect on the mean longevity of *Q. mendeli* (*F* = 22.261, *df* = 2, *p* < 0.01), and the details are shown in Table 1. Feeding *Q. mendeli* with honey solution prolonged its mean longevity to 4.80 ± 0.65 days, ranging from 4–7 days. This diet could extend longevity of this parasitoid to a greater extent than other diets, which maintained a lifespan of only 1.5 days. The study on survival pattern showed that adult female *Q. mendeli* fed with honey solution had a survival period ranging from 1–7 days, with an estimated 50% survival period of 3 days (Fig. 1). Survival periods for females supplied with pure water and no-diet were shorter, ranging from 1–2 days, with an estimated 50% survival period of only 1 day.

**Table 1.** Longevity of female *Quadrastichus mendeli* fed on different diets.

Diet	Longevity (days)
No diet	1.5 ± 0.5^a^
Pure water	1.5 ± 0.5^a^
Honey solution	4.80 ± 0.65^b^

Values are shown as mean ± *SE*; *F* = 22.261, *df* = 2, *p* < 0.01.

Means followed by the same superscript letter within each column are not significantly different (Tukey’s honestly significant difference test, *p* < 0.05).

### Fecundity of *Q. mendeli*

#### Potential fecundity

**Figure 2.**
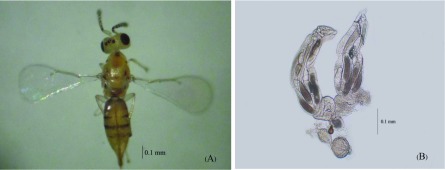
*Quadrastichus mendeli*; (A) adult female, and (B) mature eggs in ovary.

**Figure 3.**
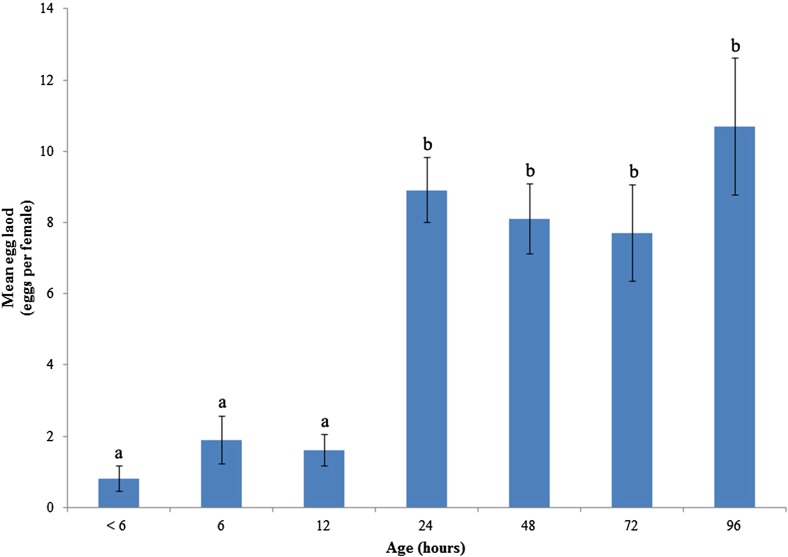
Effect of age on mean egg load of *Quadrastichus mendeli* (*F* = 14.48; *df* = 6; *p* < 0.01). Vertical bars indicate standard error.

Adult female *Q. mendeli* are shown in Figure 2A and mature eggs in ovary in Figure 2B. Dissection of the female *Q. mendeli* ovaries at < 6, 6, 12, 24, 48, 72, and 96 h after emergence revealed that all the eggs of newly emerged females (<6 hour-old) were immature. However, some were mature when the female had reached 6 h of age after emergence. Mature eggs are elongated with a long stalk of 0.4–0.6 mm in length. The average potential fecundity (egg load) of females, aged < 6, 6, 12, 24, 48, 72, and 96 h was 0.8 ± 0.36, 1.9 ± 0.67, 1.6 ± 0.45, 8.9 ± 0.91, 8.1 ± 0.97, 7.7 ± 1.36, and 10.7 ± 1.92 eggs per female, respectively. The mean egg load in all ages was 8.85 ± 0.67 eggs per female, ranging from 3–18 eggs per female. The effect of female age (hour after emergence) on the mean egg load of *Q. mendeli* is shown in Figure 3. One-way analysis of variance showed that age had a highly significant effect on the mean egg load of this parasitoid (*F* = 14.48; *df* = 6; *p* < 0.01).

**Figure 4.**
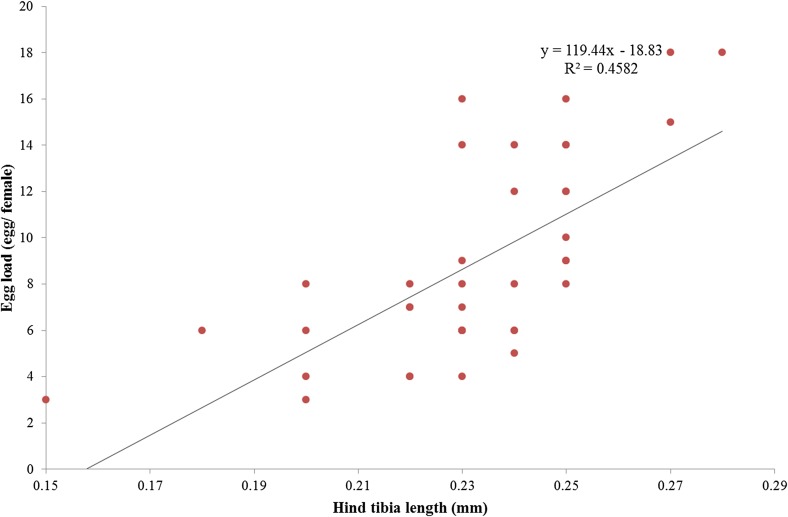
Relationship between predicted egg load (*y*) and size (*x*) of *Quadrastichus mendeli* (*n* = 40, *p* < 0.01).

By using the length of the hind tibia as a substitute for female size, the average size of female *Q. mendeli* in all ages was 0.23 ± 0.003 mm, ranging from 0.15 to 0.28 mm. The results showed a positive relationship between egg load (*y*) and size (*x*) (*y* = 119.44*x*–18.83, *R* ^2^ = 0.458, *n* = 40, *p* < 0.01) (Fig. 4). The coefficient of regression (*R* ^2^) indicated that size influenced egg loads (number of mature eggs) at the level of 45.80%.

#### Realized fecundity

Determination of the appropriate gall stage for the parasitism of *Q. mendeli* showed that the females parasitized both young and mature larvae of *L. invasa* (12–33 days after *L. invasa* oviposition). This result revealed that the mean realized fecundity of female *Q. mendeli* throughout her life span was 2.47 ± 0.30 progenies per female, ranging from 1–7 progenies per female.

### Developmental time of *Q. mendeli*

**Figure 5.**
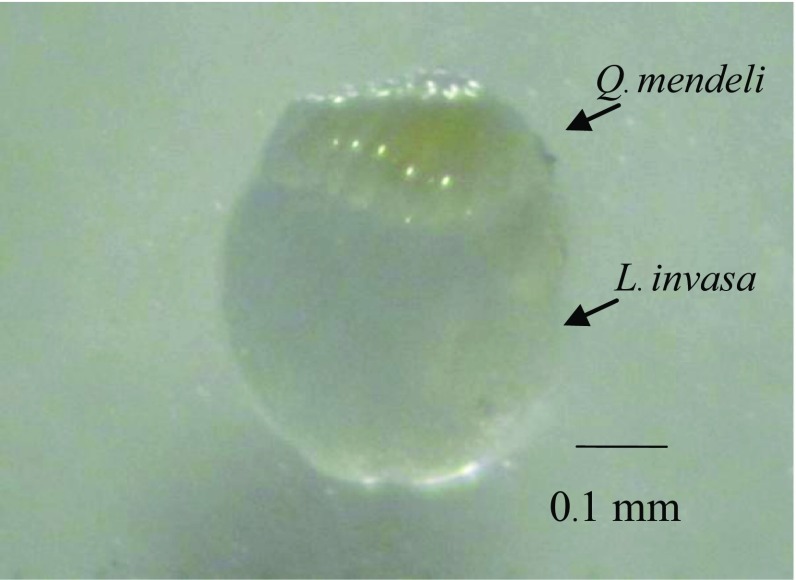
Larva of *Quadrastichus mendeli* developing on that of *Leptocybe invasa.*

**Figure 6.**
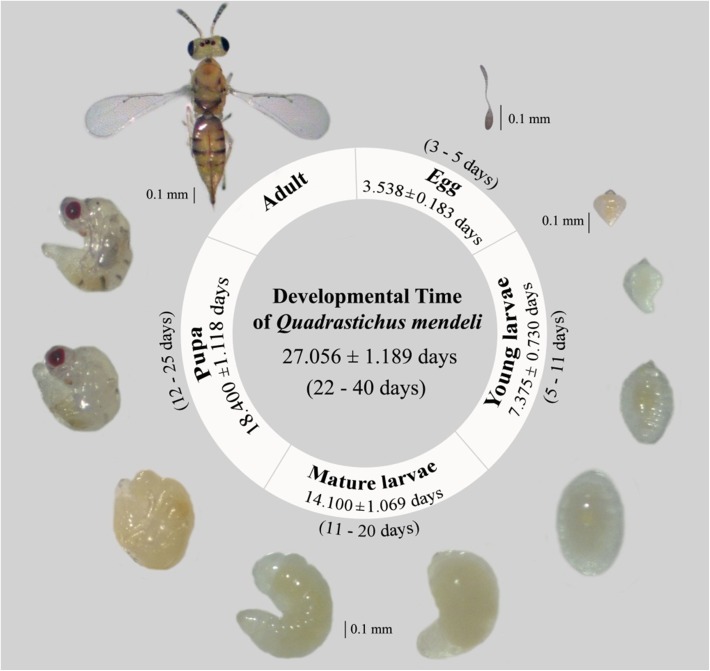
Development of *Quadrastichus mendeli*. Values are shown as mean ± *SE*; range is shown in parentheses = (minimum–maximum).

The single egg of *Q. mendeli* developed as a solitary ectoparasitoid and completed its development outside the host body (Fig. 5). The mean developmental time of *Q. mendeli*, from egg to adult stage, was 27.06 ± 1.19 days, ranging from 22 to 40 days. The development of egg, young larval, mature larval, and pupal stage took 3.54 ± 0.18, 7.38 ± 0.73, 14.10 ± 1.07, and 18.40 ± 1.12 days, respectively. Young larvae are droplet or oval-shaped, while mature larvae are C-shaped (Fig. 6).

## Discussion

*Leptocybe invasa* is a thelytokous species [32, 36, 54]. Males of *L. invasa* have however been recorded in many countries. In Thailand, the mean sex ratio between the female and male offspring of *L. invasa* in laboratory test was 1.97:1, but the males were non-functional [43]. The presence of males was directly related to infection of *Rickettsia* in its reproductive tissues [24, 36, 50], and led to a difference in the *L. invasa* sex ratio. Regarding the sex distortion of offspring, the *L. invasa* population in Thailand belonged to the Chinese lineage. However, data from a mitochondrial study indicated that the *L. invasa* populations in Thailand were a Western and Chinese lineage combination [11].

*Quadrastichus mendeli* is a uniparental species [28, 52]. It is a solitary ectoparasitoid wasp. The reproductive mode of *Q. mendeli* is thelytoky [21, 28] which was manipulated by infection of *Rickettsia* [21]. The biological traits of *Q. mendeli* at 27 ± 1 °C in this study showed that some findings differed from those at 25 °C in Israel [28]. In particular, the estimated 50% survival rate and the mean developmental time of *Q. mendeli* in this study (3 days and 27.06 days) were shorter than those in Israel (6 days and 30 days) [28]. The findings varied considerably, probably due to differences in methodologies and experimental condition settings. Temperature is an important environmental factor affecting growth and development of insects [55].

The longevity, potential and realized fecundity, and developmental time of *Q. mendeli* in this study were lower than those of *L. invasa* [43]. Comparing the mean developmental time of *Q. mendeli* (27.06 days) with that of *L. invasa* (45.96 days) [43], it was found that the short life cycle of *Q. mendeli* could be considered useful for this parasitoid, while other traits (i.e. longevity, fecundity) were not as favorable. With a short life cycle in a parasitoid, the species can produce its progeny at high numbers of generations per year and this plays a part in increasing the population growth rate [19, 20, 44]. However, the presence of males (although not functional) in *L. invasa* offspring may reduce the probability of parasitization of the next generation of *Q. mendeli*. There is evidence that the short lifespan of *Closterocerus chamaeleon* could play a role in the successful control of the eucalyptus gall wasp *Ophelimus maskelli* in Israel [38]. Thus, the uniparental nature of *Q. mendeli* and its shorter developmental time in comparison to its host can be considered good reasons for its potential use to minimize damage caused by *L. invasa* in Thailand.
